# Again

**Published:** 1874-03

**Authors:** 


### AGAIN.

O, SWEET and fair ; O, rich and rare,
  That day so long ago,
The autumn sunshine everywhere,
  The heather all aglow;
The ferns were clad in cloth of gold,
  The waves sang on the shore;
Such suns will shine, such waves will sing
  For ever, evermore.

O, fit and few; O, tried and true
  The friends who met that day;
Each one the other's spirit knew,
  And so in earnest play
The hours flew past, until at last
  The twilight kissed the shore;
We said, "Such days shall come again
  For ever, evermore."

One day again, no cloud of pain
  A shadow o'er us cast,
And yet we strove in vain, in vain
  To conjure up the past;
Like, but unlike the sun that shone,
  The waves that beat the shore,
The words we said, the songs we sung,
  Like—unlike—evermore;

For ghosts unseen crept in between,
  And, when our songs flowed free,
Sang discords in an undertone,
  And marred the harmony.
" The past is ours, not yours," they said,
  " The waves that beat the shore,
Though like the same, are not the same,
  O, never—never more!"

